# Early-Stage Glottic Squamous Cell Carcinoma in the Era of Image-Guided Radiotherapy

**DOI:** 10.3389/fonc.2021.753908

**Published:** 2021-09-20

**Authors:** Amit Gupta, Kee Howe Wong, Kate Newbold, Shreerang Bhide, Chris Nutting, Kevin Joseph Harrington

**Affiliations:** ^1^Radiotherapy and Imaging, The Royal Marsden NHS Foundation Trust and the Institute of Cancer Research, London, United Kingdom; ^2^Head and Neck Department, The Royal Marsden NHS Foundation Trust, London, United Kingdom

**Keywords:** larynx cancer, radiotherapy, vocal cords, MR-guided radiotherapy, 3D- conformal radiotherapy

## Abstract

Early-stage squamous cell cancer (SCC) of the glottis has a good prognosis. Therefore, patients have long survival outcomes and may potentially suffer from late toxicities of radiotherapy. Radiotherapy with a conventional parallel-opposed-pair or anterior-oblique beam arrangements for stage 1 and 2 glottic SCC have field borders that traditionally cover the entire larynx, exposing organs-at-risk (e.g. carotid arteries, contralateral vocal cord, contralateral arytenoid and inferior pharyngeal constrictor muscles) to high radiation doses. The potential long-term risk of cerebrovascular events has attracted much attention to the dose that carotid arteries receive. Swallow and respiratory motion of laryngeal structures has been an important factor that previously limited reduction of the radiation treatment volume. Motion has been evaluated using multiple imaging modalities and this information has been used to calculate PTV margins for generation of more limited target volumes. This review discusses the current literature surrounding dose-effect relationships for various organs-at-risk and the late toxicities that are associated with them. This article also reviews the currently available data and effects of laryngeal motions on dosimetry to the primary target. We also review the current limitations and benefits of a more targeted approach of radiotherapy for early-stage glottic SCCs and the evolution of CT-based IGRT and MR-guided radiotherapy techniques that may facilitate a shift away from a conventional 3D-conformal radiotherapy approach.

## Introduction

Squamous cell cancer (SCC) represents the most common invasive neoplasm of the larynx (1). SCCs arising from the glottic subregions of the larynx are deemed low-risk for regional or distant metastasis due to the meagre vocal cord lymphatic drainage system (2). Supraglottic and subglottic cancers are less common, accounting for up to 35% and 4% of all laryngeal cancers respectively (3). Early-stage supraglottic and subglottic cancers may be more locally infiltrative and have a higher risk of lymph node metastases than glottic SCCs (4). However, management of early-stage (T1-T2/N0) glottic and subglottic SCCs remains similar (5, 6).

The treatment paradigm for early-stage glottic SCC (ESGC) has remained unaltered for many decades. The two main treatment options are laser microsurgery or radical radiotherapy, both of which have similar outcomes in terms of local disease control (7). Despite advances in radiotherapy delivery methods, such as intensity-modulated radiotherapy (IMRT), the radiotherapy technique for ESGCs has not changed. 3D-conformal radiotherapy (3D-CRT) and generous clinical target volumes (CTV) are still the mainstay of management, particularly within the United Kingdom (8).

In the recent past, image-guided radiotherapy (IGRT) has evolved with the advent of on-set cone-beam CT (CBCT) imaging and, more recently, the introduction of the MR-linear accelerator (MRL) that provides real-time intra-fraction dynamic soft tissue data. In ESGC, a “conventional” radiotherapy beam arrangement is still used to cover the tumour and a large volume of surrounding non-target tissue as laryngeal motion remains a concern. As the precision of radiotherapy delivery techniques continue to improve, there is a push for laryngeal radiotherapy to evolve in parallel. This paper reviews the historical context and potential shortcomings of conventional radiotherapy techniques and introduces the future concepts of adaptive radiotherapy, specifically in the context of ESGCs.

## Patterns of Disease Spread

Kirchner originally published a pathological analysis of 200 partial/total laryngectomy specimens, 52 of which had glottic SCCs, and showed patterns of disease spread that were common between specimens (9). ESGCs are largely bound by surrounding fibroelastic membranes. T1 lesions are bound by the conus elasticus, which forms a firm barrier against tumour growth. Spread beyond the vocal cord frequently occurs through invasion of the anterior commissure, as the attachment of Broyles’ ligament onto the thyroid cartilage serves as a weak point, where tumour may invade through the cricothyroid membrane into the laryngeal cartilage and supraglottic or subglottic spaces (10). Anterior commissure invasion appears to be the most important prognostic determinant for local control for ESGC (11).

Invasion of cancer to a point where vocal cord mobility is impaired (T2) is either caused by invasion of the thyroarytenoid muscle or spread along the mucosal surface of the cord itself. Complete replacement of the thyroarytenoid muscle is the most frequent prerequisite before developing complete vocal cord fixation and spread. Less frequent causes of impaired vocal cord mobility/fixation include subglottic extension (T2) or direct extension along the upper surface of the cord into the thyroid cartilage (T3). Glottic SCCs are slow growing and approximately two thirds of tumours are contained at the level of the glottis at diagnosis (12). In contrast, subglottic tumours are more locally invasive, have higher rates of submucosal infiltration and fibroelastic barriers that are more susceptible to penetration (4).

## Conventional Radiotherapy

**Figure 1:**
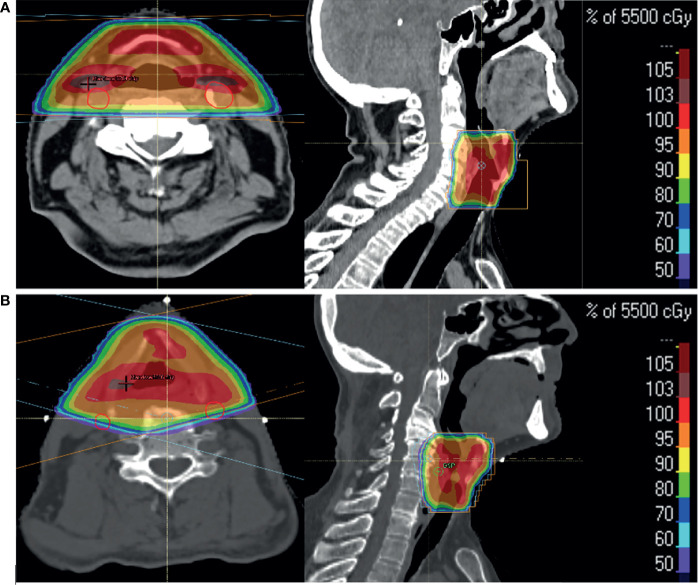
Two patients with T1a glottic SCC treated with radical radiotherapy to a dose of 55 Gy in 20 fractions over 4 weeks. Axial and sagittal images show percentage dose coverage from a bilateral parallel-opposed **(A)** and anterior-oblique **(B)** beam arrangement. Carotid arteries (red) highlight their proximity to the primary targets and doses received.

Other than transoral laser microsurgery, for stage T1a, ESGCs and subglottic tumours are conventionally treated with a beam arrangement that includes either two lateral parallel-opposed or two anterior-oblique fields, (**Figure 1**) (13). Field sizes for ESGCs are typically 25 to 36 cm^2^, (for T1 and T2 respectively). Field borders typically cover the entire larynx as there is uncertainty in the degree of motion and pattern of local tumour spread (14). For ESGC, elective nodal irradiation for N0 disease is not routinely performed, although the evidence base for subglottic cancers remains weak. Uncertainties in the local extent of tumour can arise from lack of direct visualisation of tumour growth (particularly the inferior surface of the vocal cords) or inadequacies in imaging modalities that make gross tumour volume (GTV) identification difficult (15).

Decisions between laryngeal-preserving or non-preserving treatments largely depend on the presence of thyroid cartilage invasion. CT-based imaging is the primary modality for staging laryngeal tumours because it has been deemed more sensitive at detecting cartilage invasion (quoted between 80-100%) (16). However, the specificity of CT and MRI for cartilage invasion is similar (approximately 70-80%) and both can overestimate cartilage invasion (17), although this has been improved with dynamic contrast-enhancement studies or dual-energy CTs (18, 19). CT and MRI together have a high negative-predictive value for cartilage invasion and MRI particularly can be a more helpful modality for early-stage tumours where soft tissue assessment of the vocal cord or anterior commissure is necessary (20).

The varying degrees of cartilage ossification can present challenges when trying to distinguish thyroid cartilage from tumour on CT (21). Thyroid cartilage calcification and air can impede visualisation of laryngeal structures using ultrasound (22). However, a prospective study compared the performance of ultrasound against CT imaging in assessments of tumour invasion (23). There was a trend towards improved sensitivity in the detection of thyroid cartilage invasion when using ultrasound (87.5%) compared to CT (75%). A statistically significant improvement in the specificity of detection of paraglottic space invasion was seen with ultrasound. Dhoot et al. performed a similar study but with pathological confirmation of thyroid cartilage invasion post-laryngectomy (24). A trend towards improved sensitivity in detection of thyroid cartilage invasion was also noted for ultrasound (98%) compared to CT (91%).

With the advances in imaging technology and protocols, definition of tumour extent has improved to a point where it is possible to avoid conventional radiotherapy beam arrangements and delivery of 100% dose to large CTV and Planning Target Volumes (PTV).

## Dose-Volume Effects to Organs-at-Risk

### Deglutition

The muscles involved in the complex process of deglutition have recently been defined into groups that comprise 7 different “functional swallowing units” (25). The muscles that can be damaged with resulting long-term dysphagia and aspiration post-radiotherapy were described by Eisbruch et al. (26). Based on video-fluoroscopy (VF) and CT-based analyses, they quantified the degree of functional and anatomical changes in the muscle groups after radiotherapy (with or without concurrent chemotherapy) for various head and neck cancers (HNC). These muscles are termed as “dysphagia-aspiration-related structures – DARS.”

The pharyngeal constrictor muscles (superior (SPCM), middle (MPCM) and inferior (IPCM)), the floor of mouth (geniohyoid, mylohyoid and anterior digastric) and laryngeal (thyroarytenoid, cricoarytenoid and supraglottic aryepiglottic) muscles were all identified as key components of the different stages of deglutition (27). Although peak dysfunctions in global deglutition are observed around 3 months post-completion of radiotherapy, muscle groups involved in different phases of deglutition show variable extents of recovery at 12 months (28).

Subsequent studies have shown positively correlating dose-effect relationships for different muscle groups and resultant chronic functional and anatomical changes in swallowing (29–32). Methods of assessment of functional dysphagia have varied between studies, but pooled analyses, mainly consisting of heterogeneous retrospective studies, suggest that for a significant reduction in chronic dysphagia and aspiration risk, the mean pharyngeal constrictor dose of <50 Gy (at 2 Gy per fraction) should be the target (33, 34). Caglar et al. and Caudell et al. analysed specific relationships between dose to laryngeal or IPCM structures and gastrostomy dependency (35, 36). They concluded that more than 50 Gy to larynx and IPCM was a significant risk factor for aspiration, stricture and chronic gastrostomy dependency. Accordingly, SPCM irradiation was significant in the context of radiotherapy for oropharyngeal and nasopharyngeal SCCs.

A multi-centre randomised phase 3 trial investigated dysphagia-optimised IMRT (DO-IMRT) to spare DARS for 112 patients with oropharyngeal and hypopharyngeal cancers (37). Mandatory dose constraints were set as Dmean <50 Gy for all pharyngeal constrictor muscles. They were the first to demonstrate that the MD Anderson Dysphagia Inventory score was improved with DO-IMRT compared to standard IMRT.

3D-CRT for ESGCs treats significant volumes of IPCM and supraglottic larynx. 3D-CRT has remained the method of treatment at our institute to date. Analyses of the dose statistics to organs-at-risk (OARs) within our patient cohort, who receive 55 Gy in 20 fractions over 4 weeks, are shown in **Table 1** (unpublished data).

**Table 1:** Dose statistics analysed for 32 patients treated with 3D-CRT at Royal Marsden Hospital, Sutton.

OARs	3D-CRT (n = 32)		Parrallel-opposed-pair (n = 22)		Anterior-oblique (n = 10)	
	Dmean (SD), Gy	Dmax to 1cm^3^ (SD, Gy)	Dmean (SD), Gy	Dmax to 1cm^3^ (SD, Gy)	Dmean (SD), Gy	Dmax to 1cm^3^ (SD, Gy)
Carotid (ipsilateral)	45.8 (4.39)	57.5 (0.76)	47.11 (3.28)	57.58 (0.77)	42.89 (5.27)	57.35 (0.76)
Carotid (contralateral)	45.44 (0.4.88)	57.39 (0.89)	46.79 (3.92)	57.44 (0.92)	42.48 (5.64)	57.28 (0.84)
Arytenoid (ipsilateral)	55.95 (1.13)	-	55.91 (1.29)	-	56.04 (0.72)	-
Arytenoid (contralateral)	56.08 (0.74)	-	56.07 (0.81)	-	56.11 (0.62)	-
IPCM	54.82 (0.99)	-	54.61 (0.97)	-	55.27 (0.92)	-
Thyroid Gland	26.88 (8.82)	-	25.55 (8.77)	-	29.80 (8.63)	-

Carotid arteries were contoured 1 cm superior and inferior to the primary PTV. Mean (Dmean) and maximum (Dmax) doses are shown for serial OARs. All OARs were expanded by 3 mm to a “Planning Risk Volume.” Dose statistics are also shown separately for parallel-opposed-pair and anterior-oblique beam arrangements.

A hypothesis-generating prospective analysis of a small population of patients with largely oropharyngeal SCCs, with no laryngeal invasion, demonstrated that over 65 Gy delivered to the aryepiglottic folds had the strongest correlation between dose and risk of aspiration (38). Studies have generally pooled data from patients with a variety of primary HNCs who have largely received IMRT with or without concurrent chemotherapy. Thus, it has been difficult to deduce precise dose-effect relationships for individual laryngeal structures, including their relative contributions towards the degree of dysphagia.

### Voice

Voice relies on the function of multiple organ systems, from the lungs to individual components within the larynx, all working in concert with the oral cavity and pharynx to create speech (39). Assessment of voice entails a variety of objective and subjective methods that appraise all aspects. Historically, there have been contradictory reports on the longer-term outcomes of radiotherapy for ESGCs (40). However, studies performed in the last two decades have adopted more sophisticated tests for analysis of voice and reported more consistent outcomes.

The impact of accelerated hypofractionated radiotherapy on voice quality was demonstrated in a prospective study of 25 patients with ESGC (41). When compared to healthy controls, baseline voice quality, measured using serial electroglottographic and acoustic analysis methods, was worse but improved by 12 months post-radiotherapy. However, voice quality still remained significantly worse than compared to the normal subjects.

Fung et al. compared the differences in the impact of 3D-CRT on voice in a small number of patients (n=17) who received radiotherapy to non-laryngeal SCC (60-74 Gy in 30-37 fractions, average laryngeal dose 50 Gy) and stage T1a glottis SCC (61 Gy in 25 fractions) (42). They used a variety of objective and subjective methods to analyse voice and showed evidence of global vocal dysfunction that was greater in patients treated for non-laryngeal wide-field radiotherapy. However, harmonic-noise ratio (HNR) and shimmer were worse in the T1a glottis patients, a finding also noted by Mekis et al. (43). HNR and shimmer are voice amplitude-based measures and affected by the structural integrity of the vocal cords. These studies suggest that voice/speech is dependent on multiple factors such as extra-laryngeal muscle and salivary gland functions as well as chronic radiotherapy-related changes such as fibrosis or lymphatic drainage, which alter the physical structures of the upper airway tract.

A mixture of retrospective and prospective studies involving ESGCs have all shown subjective and objective improvements in all parameters of voice within 12 months of radical 3D-CRT (44–49). Despite subjective improvements, objective measures of voice do not return to population-derived normal ranges (42, 48, 50). Three studies all compared voice function following radiotherapy against patients having transoral laser microsurgery (45–47). There are conflicting outcomes between the two treatment modalities, which is the reason for the lack of conclusive evidence to suggest either treatment option being superior for improvements in short-term voice quality. Arias et al. also showed that Voice Handicap Index and quality of life (QoL) measures were superior with radiotherapy compared to surgery. Improvements in QoL are consistent findings across the majority of studies, suggesting that, despite persistent objective deficits in voice function, patients feel their communicative functions are satisfactory.

Patients with ESGCs generally have good prognoses and delayed effects of treatments are, therefore, more likely to impact on later QoL. Hocevar-Biltezar et al. and Ma et al. performed studies that followed up patients for over 1 year after radical radiotherapy for T1 glottic SCC (51, 52). Direct visualisation revealed morphological defects of varying severities in over 95% of patients with contralateral vocal cord changes in 50% of cases. Evolution of chronic radiotherapy-related changes demonstrated worsening of dysphonia, speech loudness and fatigue.

Fu et al. evaluated the degree of laryngeal oedema in patients mainly with T1-2 SCCs receiving between 50-80 Gy in 1.5-1.8 Gy per fraction, with conventional radiotherapy beam arrangements (53). Roughly 15% of patients had laryngeal oedema lasting over 3 months post-radiotherapy. 11.7% of patients with no proven disease recurrence had oedema that resolved within 2 years, with smoking and alcohol being associated with more severe and longer lasting oedema. Doses above 70 Gy, larger field sizes and more advanced disease were associated with more severe oedema. This would be expected adversely to affect quality of voice and it has been suggested that dose to non-involved laryngeal tissue should be maintained below 40-45 Gy to maintain function (54). Furthermore, speech-related QoL outcomes were significantly associated with doses above 66 Gy to the aryepiglottic folds, pre-epiglottic space, false cords or lateral pharyngeal walls (55).

### Carotid Arteries

The left and right common carotid arteries pass posterior to the respective sternoclavicular joints before they ascend the neck and bifurcate into external and internal branches at the level of the superior border of the thyroid cartilage (56). The internal carotid artery extends towards the base of skull, whereas the external carotid artery further divides into 8 branches that supply the extracranial head and neck regions (57).

Radiotherapy plans for locally-advanced HNCs inevitably treat a significant length of the carotid arteries when neck nodes are treated. The chronic sequelae of carotid irradiation have been well described in the literature. Chung et al. retrospectively demonstrated carotid wall thickening visible on MRI as early as 6 months post-irradiation (58). Patients were treated for a range of HNC primary sites using conventional 3D-CRT techniques to cover neck nodes, with doses ranging between 40-74 Gy.

Histopathological analyses of irradiated carotid arteries in cadavers have provided insights into the pathophysiological changes that cause carotid artery stenosis (56). The sequence of events that cause chronic vessel changes are analogous to the formation of atherosclerosis, as would be seen from prolonged exposure to the Framingham risk factors (59). However, acute changes have been explored in irradiated canine femoral vessels, that showed endothelial damage as early as 48 hours post-exposure, followed by destruction of the internal elastic lamina and endothelial thickening by 1 week post-exposure. By 4 months post-exposure there was focal necrosis and fibrosis of the media, accompanied by chronic inflammation and minimal thrombosis of the adventitia. The combination of chronic fibrosis and atherosclerosis, caused by inflammation and smooth muscle migration and foam cell generation, is what results in narrowing of the lumen (60). Damage and necrosis of the vasa vasorum is a distinguishing feature of radiotherapy-induced adventitial damage (61, 62).

The effects seen in medium- to large-sized arteries are similar in appearance and are limited to irradiated regions. Although intima-media thickness (IMT) and carotid artery lumen narrowing is readily visible on carotid ultrasound scans by 6 months post-irradiation, there is little impact on blood flow (63, 64). However, carotid artery stenosis and the risk of developing associated symptoms progressively worsens, with Cheng et al. observing an annualised rate of progression from <50% to ≥50% stenosis of 15.4%, compared to 4.8% in non-irradiated arteries (65). An early case-controlled series of patients under 60 years of age, who received radical-dose radiotherapy (60-66 Gy in 2-2.4 Gy per fraction) for ESGC, were found to have a median interval to stroke event of 10.9 years and absolute excess risk of 3.8 strokes (per 1000 patients/year) (66). The risk of a carotid stenosis-related event (e.g. cerebrovascular accident (CVA), transient ischaemic attack, amaurosis fugax) rises at an exponential rate after 10 years post-exposure (67, 68). Many studies have since demonstrated significantly enhanced carotid artery stenosis as a result of exposure to radiotherapy (69–73).

As a result, much effort has been exerted in deviating away from conventional lateral parallel-opposed pair beam arrangements for ESGC to alternative planning techniques such as IMRT or volumetric arc therapy (VMAT). Garcez et al. demonstrated a significant reduction in maximum and mean carotid artery dose by switching from a lateral parallel-opposed pair (POP) to an anterior-oblique beam arrangement (74). With dose constraints placed on carotid arteries, IMRT techniques can achieve substantial dose reduction compared to 3D-CRT techniques (75–78). PTV coverage with IMRT or VMAT has better conformity and heterogeneity indices compared to 3D-CRT techniques (75). Although spinal cord dose tends to be higher for IMRT, doses do not approach organ tolerance.

There is much heterogeneity between studies, variations in primary tumour site, dose, radiotherapy delivery techniques and inconsistent dosimetry reporting, making it difficult to establish any dose-effect relationships between carotid artery dose and clinical consequences. A single cross-sectional study of 14 patients who had single-side neck irradiation showed that IMT was significantly different for doses above 35 Gy only (79). Studies have since used V35 and V50 to report carotid artery doses in IMRT planning (80). More recently a retrospective analysis of 750 patients who received radical dose radiotherapy for HNC demonstrated the absolute volume of carotid artery, particularly the common carotid and bifurcation, that received 10 Gy or greater was the most important prognostic factor for an ischaemic CVA (81). However, this study is limited by the relatively short mean follow-up time of 3.4 years.

Rosenthal et al. initially described their 3-field IMRT protocol for treating T1-T2 glottic SCCs to doses ranging from 63-65.25 Gy in 2.25 Gy per fraction to 66-70 Gy in 2 Gy per fraction (77). CTV was contoured to cover the whole larynx, as for conventional 3D-CRT methods. Carotid artery doses were kept as low as possible but V35 and V50 were recorded for each patient. A follow-up paper reported outcomes for 215 patients treated with either lateral POP (153 patients) or IMRT (62 patients) for T1 disease only (78). There were no statistically significant differences in 3- or 5-year local control or overall survival rates for patients with T1a/b disease or between the two radiotherapy modalities, a finding mirrored by Zumsteg et al. (82). Age <60 years and performance status ≤1 were the only factors that correlated with improved overall survival on univariate and multivariate analyses. Four CVA events occurred in the conventional radiotherapy group and none in the IMRT group. Follow-up period is not long enough reliably to compare long-term effects but, based on recurrence data after a median follow-up period of 68 months, the MD Anderson Cancer Centre group now use IMRT techniques to treat all their T1 glottis SCCs.

### Laryngeal Motion

Patient set-up correction protocols would traditionally have utilised patient alignment or couch shifts using bony anatomy, which is readily visible on portal imaging systems (83). Position verification using cervical vertebrae was used under the assumption that spatial relations between bone and soft tissues are fixed. Early studies of the different components involved in deglutition utilising cineradiography showed marked differences in the ranges and directions of motions between structures (84). This was further replicated using VF, where they not only showed that laryngeal and hyoid motion was separate, but also that it was consistent regardless of the size of bolus being swallowed (85). The importance of understanding motion was highlighted when it was shown that local control rates after radiotherapy delivered by matching to vertebral anatomy were worse than when using laryngeal cartilages (86).

Hamlet et al. more accurately quantified laryngeal motion in patients with early-stage laryngeal cancers using VF (87). They demonstrated that laryngeal motion was greatest in the cranio-caudal (CC) (mean 25.5 mm), more limited in anterior-posterior (AP) (8.3 mm) and negligible in left-right (LR) directions. Total duration of deglutition was 1 second, with resultant impact on dose reduction at the superior field edge being as little as 0.5%. Van Asselen et al. further demonstrated, using megavoltage X-ray imaging, that frequency of deglutition is highest in the first 5 fractions of radiotherapy, after which it decreases between fractions 6-10 before slowly increasing by the end of treatment, although still not becoming as frequent as during the first 5 fractions (88). With the realisation that frequency and duration of deglutition is limited, there has been much interest in evolving conventional radiotherapy techniques for ESGC. Motion has, therefore, received further in-depth exploration using more sophisticated imaging modalities such as CBCT and MRI. Bradley et al. first described the use of MR-based imaging to study motion in tumours from a range of head and neck primary subsites, including 5 patients with glottis SCC (89). CC (31.2 mm) and AP (11.6 mm) motion of vocal cords were described during deglutition. They also showed laryngeal motion up to 7.3 mm (CC) and 3.4 mm (AP) during rest, a finding mirrored by other groups (90, 91).

Anatomical and temporal definitions of when deglutition begins and ends have not been established. However, it is apparent that motion, particularly in the CC direction, is of the greatest concern amongst centres looking to decrease irradiation volumes for ESGC. Quantitative data on distance, duration and frequency do show some variation in these factors. Therefore, individual institutions have performed their own motion studies in order to establish their own PTV margins (89, 92–94). In the context of conventional CTV definition for ESGC, Bruijnen et al. deemed it unnecessary for Internal Target volume (ITV)/PTV margins to account for the short duration of laryngeal excursion during deglutition (94). Using cine-MRIs to establish respiratory-induced vocal cord motion and retrospective CBCT data to calculate systematic and random errors, they calculated non-isotropic PTV margins up to a maximum of 4.3 mm superiorly.

There has been a push towards IMRT techniques in an attempt to spare OARs and to reduce additional soft tissues receiving high radiotherapy doses. VMAT or step-and-shoot IMRT are two methods that were explored by Bahig et al. (95). When comparing both partial- and whole-larynx volumes, swallowing was found to have minimal dosimetric impact, with even further improvement when patients were asked not to swallow. Instructing patients not to swallow was felt to be a feasible strategy based on the short durations of each fraction delivered by VMAT. They also described the importance of routine image-guidance systems as they had noticed gradual laryngeal shift over the course of radiotherapy.

## Evolution of Radiotherapy for Early Stage Glottic SCCs

The wealth of individual centre-level data provides reassurance on the insignificance of laryngeal motion on dosimetry. The potential benefits of sparing additional OARs, as well as the equivalent long-term control outcomes from focused surgical laser resection have resulted in some centres exploring alternative radiotherapy techniques to smaller normal tissue and target volumes. For some centres, the already high cure rates for ESGC with conventional POP beam radiotherapy has provided little motivation to move towards IMRT-based techniques. Feigenberg et al. argued a case against IMRT, by suggesting that 3D-CRT already achieves high rates of success with under 2% long-term severe complication rates. Their own data showed an increase in salvage laryngectomy rates after IMRT for ESGC and potential for underdosing around the anterior commissure (96). Many of these points have since been addressed by subsequent studies.

### Target Definition

Eisbruch et al. described the importance of accurate target delineation and identified proper GTV outlining as having the highest reward-to-risk ratio (97). GTV delineation discrepancies or errors occur when superficial mucosal disease, something that is not readily visible on radiological imaging, is not identified during clinical examination. Accuracy of target delineation was also highlighted by Kim et al. who noted higher local recurrence rates with IMRT in patients with stage T2 glottic SCCs, likely due to inadequate delineation or CTV margins, as there is a risk of tumour extension above or below the glottis that is difficult to identify on radiological imaging (14).

Traditionally, CTVs have covered the whole larynx extending cranio-caudally from the hyoid to the cricoid and axially to cover the thyroid cartilage and arytenoids. PTV margins vary between institutions, but are generally larger in the cranio-caudal directions in order to cover laryngeal motion (98). At some institutions, shifts towards IMRT or VMAT techniques and attempts to decrease CTV and PTV are also taking place, with some centres employing multiple dose levels to dose-paint the involved vocal cord alone and whole or partial larynx separately (99). Various PTV margins in these circumstances have been based on individual-centre assessments of random or systematic errors and laryngeal motion as described earlier. Osman et al. have pioneered single vocal cord irradiation (SVCI) techniques and demonstrate abilities to deliver high-dose radiotherapy to glottic subregions, achieving additional sparing of structures such as contralateral vocal cord and arytenoids (92, 100). They have achieved this by developing stringent quality assurance within their planning and image-guided treatment delivery systems to prevent target miss.

### Radiotherapy Technique and Outcomes

Analyses of the American National Cancer Database showed that between 2004 and 2015, the use of IMRT for ESGC increased from 2% to 16%, with the majority of IMRT use being within academic institutes that are driving highly conformal radiotherapy (101). Implementation of IMRT techniques requires careful analysis of the entire workflow to avoid target miss during treatment. For example, in a VMAT planning case, patients were asked not to swallow during acquisition of the planning CT and due to the longer fraction delivery times with IMRT/VMAT, patients were coached to swallow during beam position changes or planned gaps, to prevent laryngeal motion (102).

Kim et al. showed that dose de-escalation to the uninvolved larynx and maintenance of high-dose radiotherapy to smaller laryngeal targets can achieve comparable 5-year local control rates to conventional POP radiotherapy (99). There was a trend for increased local recurrences in the T2 N0 population, which highlighted the potential consequences for not adequately appreciating the risk of local subclinical spread above or below the glottis. Although follow-up periods are short, the dosimetric advantages to OARs and comparable local control rates for IMRT and VMAT have been further demonstrated by numerous retrospective analyses (11, 103–107).

Princess Margaret Hospital have implemented a “5+5” approach to treat their stage T2 N0 disease with IMRT since 2006 (86). They changed their set-up matching protocols from cervical vertebral match to thyroid cartilage in 2008. Despite low patient numbers and limited follow-up period, local control data showed a trend towards lower local recurrence rates for IMRT with thyroid cartilage matching. 89% of all local failures in the IMRT group occurred infield and in the ipsilateral vocal cord. Updated information from that institute demonstrated that by using an accelerated fractionation schedule (66-70 Gy in 33-35 fractions over 5.5-6 weeks), they further improved their 5-year local control rates from 75% to 89% (108). No out-of-field recurrences were noted and the majority of recurrences were in the marginal region. The importance of adequate image-guidance systems was strongly emphasised.

In a further step towards highly focused radiotherapy, Erasmus Medical Centre use 4D-CT methods at planning to determine resting vocal cord position and nullify systematic error on an individual patient level (109). After setting stringent dose constraints to OARs they were able to determine optimal IMRT beam angles for SVCI, the robustness of which was tested in a subsequent planning study that simulated geometrical uncertainties due to motion, anatomical variations and random errors (110). They were able to demonstrate that a PTV margin of 2 mm was safe to maintain adequate CTV dose in 10 simulated cases. 2-year local control for stage T1a disease treated with SVCI, (66 Gy in 16 fractions over 3 weeks), was reported as 100%. When compared to whole-larynx IMRT, no deteriorations in serious late toxicities or Voice Handicap Index were described (111).

SVCI is still deemed very much an exploratory treatment method, but data from 34 patients who had hypofractionated SVCI for T1a glottis SCC demonstrated 5-year local control and disease-free survival rates of 96.8% (112). The single local recurrence was in-field and did not require salvage total laryngectomy. No severe late toxicities were reported and there were significant dose reductions to contralateral vocal cord, arytenoid and other OARs.

## Future Directions

The potential benefits of sparing OARs from excessive radiation dose should encourage the adoption of IMRT techniques to treat more focused targets in ESGC. There is convincing evidence of the ability of IMRT to deliver adequate dose in a safe and effective manner. IMRT/VMAT would be most appropriate for T1b-T2 glottic SCCs, which are at higher risk of microscopic spread to the supraglottis or subglottis. Treatment techniques such as SVCI on C-arm linear accelerators are technically feasible based on current literature and are expected to be an attractive approach to treating T1a glottic SCCs. However, motion-induced target miss will likely remain an ongoing concern. Long-term outcome data are required to truly build confidence in radiotherapy to reduced volumes. This is currently being investigated in a phase-2 trial comparing radiotherapy to vocal cord only against whole larynx (113).

Although differences in acute-onset toxicities are not apparent between 3D-CRT and IMRT, the delayed-onset toxicities remain of concern. Although delayed events, such as CVA, are difficult to investigate prospectively due to the long follow-up periods required, retrospective analyses and studies looking at surrogate markers (such as IMT) provide substantial evidence of their significant impact on survivorship.

MR-guided radiotherapy systems, particularly the MRL, provide an ideal platform for treating ESGC as soft tissue may be visualised during treatment delivery. Live-target tracking during beam-on periods and gating mechanisms are still in their infancy on the MRL, however, the feasibility of delivering MR-guided radiotherapy is rapidly becoming apparent across a variety of tumour sites (114–116). Although the technology behind MR-guided treatment delivery systems are not yet readily available across the majority of cancer centres, their promising image-guidance and live tracking tools could instil much confidence in treating all stages of ESGC.

## Author Contributions

AG was the main author of the script. All other authors provided substantial guidance with the content and feedback on the manuscript. All authors contributed to the article and approved the submitted version.

## Funding

This work was supported by the Cancer Research UK programme grants C7224/A13407, C33589/A19727 and C33589/A28284.

## Conflict of Interest

KH reports personal fees for serving as an advisory board member from MSD, AstraZeneca, Amgen, Boehringer Ingelheim, Merck Serono, Mersana, Oncolys, Pfizer, Replimmune, and Vyriad; personal fees for serving as a speaker from MSD, AstraZeneca, Amgen, Merck Serono; and honoraria from MSD, AstraZeneca, Amgen, Boehringer Ingelheim, Merck Serono, Pfizer, Replimmune, and Vyriad.

The remaining authors declare that the research was conducted in the absence of any commercial or financial relationships that could be construed as a potential conflict of interest.

## Publisher’s Note

All claims expressed in this article are solely those of the authors and do not necessarily represent those of their affiliated organizations, or those of the publisher, the editors and the reviewers. Any product that may be evaluated in this article, or claim that may be made by its manufacturer, is not guaranteed or endorsed by the publisher.
